# Bi-directional signaling by membrane-bound KitL induces proliferation and coordinates thymic endothelial cell and thymocyte expansion

**DOI:** 10.1038/s41467-018-07024-0

**Published:** 2018-11-08

**Authors:** Mario Buono, Marie-Laëtitia Thézénas, Alessandro Ceroni, Roman Fischer, Claus Nerlov

**Affiliations:** 1 MRC Molecular Hematology Unit, MRC Weatherall Institute of Molecular Medicine, John Radcliffe Hospital, University of Oxford, Oxford, OX3 9DS UK; 2 Target Discovery Institute, University of Oxford, Old Road Campus, Oxford, OX3 7FZ UK

## Abstract

The ligand for the c-Kit receptor, KitL, exists as a membrane-associated (mKitL) and a soluble form (sKitL). KitL functions outside c-Kit activation have not been identified. We show that co-culture of c-Kit– and mKitL–expressing NIH3T3 cells results in signaling through mKitL: c-Kit–bound mKitL recruits calcium-modulating cyclophilin ligand (CAML) to selectively activate Akt, leading to CREB phosphorylation, mTOR pathway activation, and increased cell proliferation. Activation of mKitL in thymic vascular endothelial cells (VECs) induces mKitL- and Akt-dependent proliferation, and genetic ablation of mKitL in thymic VECs blocks their c-Kit responsiveness and proliferation during neonatal thymic expansion. Therefore, mKitL–c-Kit form a bi-directional signaling complex that acts in the developing thymus to coordinate thymic VEC and early thymic progenitor (ETP) expansion by simultaneously promoting ETP survival and VEC proliferation. This mechanism may be relevant to both normal tissues and malignant tumors that depend on KitL–c-Kit signaling for their proliferation.

## Abstract (web summary)

c-Kit receptor–Kit ligand complex signaling is known to activate c-Kit and is essential for tissue development. Here, Buono et al. show that membrane-bound KitL signaling induces proliferation via CAML-Akt-CREB pathway activation, establishing a role for bidirectional signaling in tissue expansion.

## Introduction

The c-Kit receptor and its ligand KitL form a signaling complex that plays important roles in hematopoiesis, fertility, pigmentation, digestion, and nervous system function^1^. Furthermore, activating mutation in c-Kit is observed in several malignancies, including acute myeloid leukemia, mastocytosis, gastrointestinal stromal tumors and melanoma, and c-Kit inhibitors are being developed for cancer therapy^2^.

KitL is the only known c-Kit ligand, and exsists in both a membrane-associated (mKitL) and soluble form (sKitL). Whereas sKitL is generated through juxtamembrane proteolytic cleavage, mKitL is generated by skipping of the exon that contains the proteolytic cleavage site^3^. Genetic experiments have established that mKitL and sKitL each carry out unique physiological roles: Genetic deletion of the sKitL proteolytic cleavage site resulted in loss of mast cells from the skin and peritoneum, and increased radiosensitivity^4^. In contrast, selective mKitL ablation demonstrated that mKitL expressed by thymic vascular endothelial cells (VECs) and cortical thymic epitelieal cells (cTECs) plays an important role in the survival of c-Kit-expressing early thymic progenitors (ETPs)^5^. Importantly, upon loss of mKitL from thymic stromal cells similar decreases in the number of thymocytes, thymic epithelial cells and VECs are observed^5^, indicating the presence of homeostatic mechanisms that preserve the proportionality of thymic cell types.

During development the induction of the mouse thymus occurs around embryonic day 11.5 (e11.5), followed by diversification of cortical (cTECs) and medullary thymic epithelial cells (mTECs), and vascularization around e13.5^6,7^. The vascularized thymus expands rapidly until postnatal day 12 (P12) when it reaches its adult size^8^. Several signaling molecules, including interleukin (IL-)7, Dll4, Ccl19, Ccl25, Cxcl12, BMP4, and Wnt4, have been identified as important for the expansion and differentiation of thymocytes, whereas TEC specification involves Shh, BMP4, Fgf, and Wnt signaling^9,10^. However, little is known about how thymic VECs are specified or how thymocyte and stromal cell expansion is coordinated.

Given that mKitL depletion eliminates both c-Kit signaling in thymocyte progenitors and mKitL in thymic VECs and TECs this raised the possibility that mKitL transduces a signal upon mKitL–c-Kit interaction that promotes the expansion of mKitL-expressing cells. We therefore tested whether engagement of mKitL by c-Kit elicits signaling in mKitL-expressing cells. We find that stimulation of mKitL by cell-associated or soluble c-Kit activates the Akt/mTOR/CREB pathway and increases cell proliferation. Finally, loss of mKitL in thymic VECs decreases their perinatal proliferation. Therefore, c-Kit and mKitL constitute a bi-directional signaling complex that can coordinate cell proliferation and survival in the developing thymus.

## Results

### c-Kit signals through mKitL

**Fig. 1:**
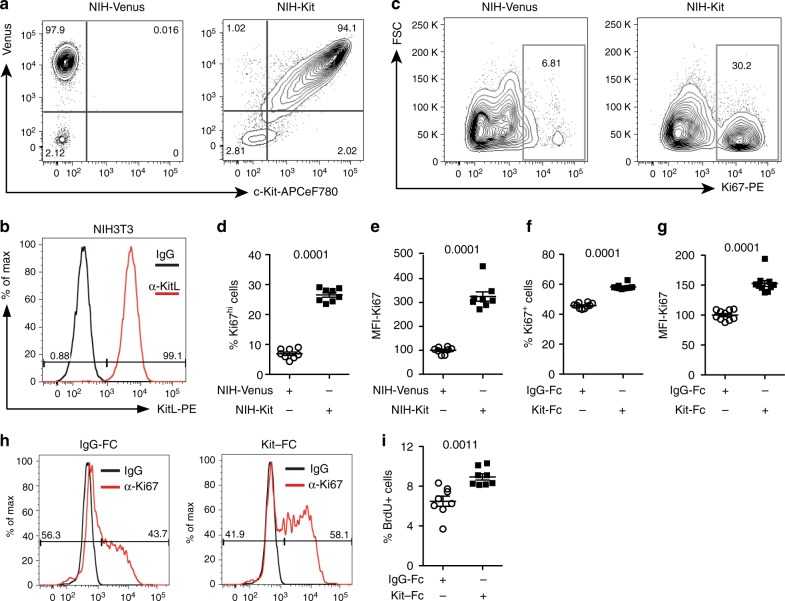
Membrane-bound Kit ligand acts as a c-Kit receptor to induce cell proliferation. **a** Flow cytometry plots of pRRL-Venus-transduced (left plot; NIH-Venus) and pRRL–c-Kit-Venus-transduced NIH3T3 cells (right plot; NIH-Kit) stained with anti–c-Kit antibody. Data are representative of three experiments. **b** Flow cytometry plot of NIH3T3 cells stained with anti-SCF antibody or control IgG, as indicated. Data are representative of three experiments. **c** Ki67 expression in NIH3T3 co-cultured together with NIH-Venus (left plot) or NIH-Kit (right plot) detected by intracellular flow cytometry. Gating shows Ki67^high^ cells. **d** Percentage of Ki67^high^ cells in cultures from **c** (*N* = 7, two experiments). Bars show mean ± s.e.m. *P*-value is shown (Student’s *t*-test). **e** Mean fluorescence intensity (MFI) of Ki67 signal in samples from **d**. Bars show mean ± s.e.m., with NIH-Venus mean = 100. *P*-value is shown (Student’s *t*-test). **f** Percentage of Ki67^+^ cells in cultures from NIH3T3 treated with 200 ng/ml Kit-Fc (*N* = 10; two experiments) or IgG-Fc (*N* = 10, two experiments), as indicated, for 24 h prior to analysis by intracellular flow cytometry. Bars show mean ± s.e.m., with NIH-Venus mean = 100. *P*- value is shown (Student’s *t*-test). **g** Mean fluorescence intensity (MFI) of Ki67 signal in NIH3T3 cells from **f**. Bars show mean ± s.e.m., with NIH-Venus mean = 100. *P*-value is shown (Student’s *t*-test). **h** Representative flow cytometric analysis of Ki67 expression in IgG-Fc (left panel) and Kit-Fc (right panel) treated NIH3T3 cells from **f**. **i** Percentage of NIH3T3 cells incorporating BrdU after 8 h treatment with 200 ng/ml IgG-Fc or Kit-Fc (*N* = 8; two experiments) as indicated. BrdU was added to the culture medium 2 h before analysis. Bars show mean ± s.e.m. *P*-value is shown (Student’s *t*-test)

**Fig. 2:**
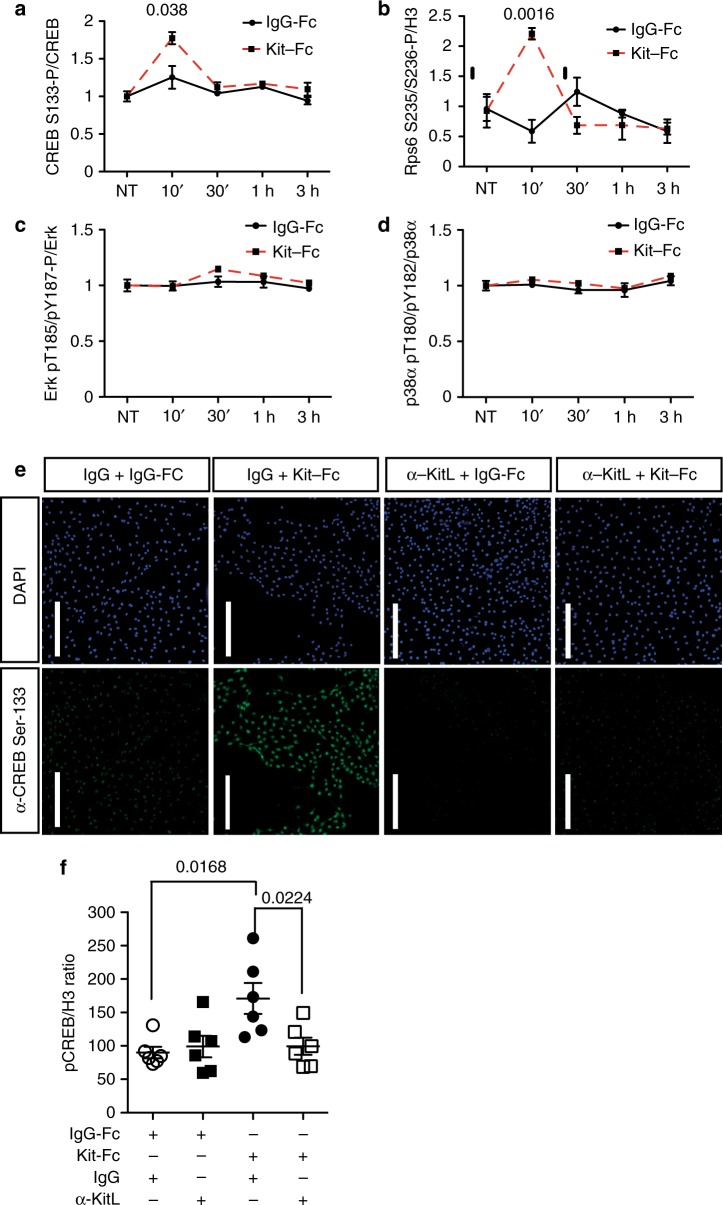
mKitL signaling induces CREB and Rps6 phosphorylation. **a** ELISA quantification of CREB S133 phosphorylation in NIH3T3 treated with 200 ng/ml IgG-Fc (*N* = 3, two experiments) or Kit-Fc (*N* = 3, two experiments) at the indicated time points. Bars show mean ± s.e.m. *P-*value is shown (Student’s *t*-test). **b** ELISA quantification of Rps6 S235/S236 phosphorylation in NIH3T3 treated with IgG-Fc (*N* = 3, two experiments) or Kit-Fc (*N* = 3, two experiments) at the indicated time points. Values represent the mean of the ratio between Rps6 S235/S236 and Histone H3 at the indicated time points, quantified by ImageJ analysis of Western blots. Bars show mean ± s.e.m. *P*-value is shown (Student’s *t*-test). **c** ELISA quantification of Erk1 T202/Y204 + Erk2 T185/Y187 phosphorylation in NIH3T3 treated with IgG-Fc (*N* = 3, two experiments) or Kit-Fc (*N* = 3, two experiments) at the indicated time points, quantified by ImageJ analysis of western blots. Bars show mean ± s.e.m. No statistically significant differences were observed. **d** ELISA quantification of p38α T180/Y182 phosphorylation in NIH3T3 treated with IgG-Fc (*N* = 3, two experiments) or Kit-Fc (*N* = 3, two experiments) at the indicated time points, quantified by ImageJ analysis of western blots. Bars show mean ± s.e.m. No statistically significant differences were observed. **e** Immunofluorescence analysis of NIH3T3 cells treated as in **i** showing CREB phospho-S133 (green) and DNA (blue, visualized with DAPI). Scale bars: 75 μm. Data are representative of three experiments. **f** Quantification of CREB S133 phosphorylation in NIH3T3 cells pre-treated with goat-IgG (g-IgG) or goat anti-KitL antibody (α-KitL) for 1 h, and treated IgG-Fc (*N* = 4, two experiments) or Kit-Fc (*N* = 6, two experiments) for 10 min. Values represent the mean of the ratio between of CREB phospho-S133 and Histone H3, quantified by ImageJ analysis of western blots. Bars show mean ± s.e.m. *P*-value is shown (Student’s *t*-test)

To test the hypothesis that mKitL has signaling capacity we expressed c-Kit in NIH3T3 cells by lentiviral transduction (Fig. 1a), generating NIH-Kit cells. Upon co-culture of NIH-Kit cells with wild-type NIH3T3 cells, where mKitL is endogenously present (Fig. 1b), we observed a strong upregulation of the Ki67 proliferation marker in the wild-type NIH3T3 cells, not observed upon co-culture with NIH3T3 cells transduced with the control Venus expression vector (NIH-Venus) (Fig. 1c–e; Supplementary Fig 1). Inhibition of c-Kit signaling with Imatinib did not decrease proliferation of NIH3T3 cell in NIH-Kit co-cultures, indicating that c-Kit activation in NIH-Kit cells did not indirectly contribute to NIH3T3 proliferation (Supplementary Fig 2a–c). This was supported by the ability of NIH3T3 cells expressing kinase-dead c-Kit (NIH-KitK623M cells)^11^ to induce proliferation similarly to NIH-Kit cells (Supplementary Fig 2d–f). Incubation of NIH3T3 cells with a recombinant fusion between the c-Kit extracellular domain (ECD) and the immunoglobulin G (IgG) constant domain (Kit-Fc) also promoted cell cycle progression, as measured by Ki67 expression (Fig. 1f–h) and bromodeoxyuridine (BrdU) incorporation (Fig. 1i). These results demonstrated that exposure of mKitL-expressing NIH3T3 cells to the c-Kit ECD, either presented on a neighboring cell surface or in solution, is sufficient to elicit a proliferative response. We screened major signaling pathways for activation downstream of mKitL, and observed that incubation with Kit-Fc induced transient serine 133 (S133) phosphorylation of CREB (Fig. 2a) and serine 235/236 (S235/S236) phosphorylation of Rps6 (Fig. 2b) in NIH3T3 cells, whereas activating phosphorylation of Erk1/2 or p38α was not observed (Fig. 2c, d). CREB S133 phosphorylation was accompanied by nuclear accumulation of CREB phospho-S133 (Fig. 2e). A blocking antibody against mKitL abolished Kit-Fc-induced CREB S133 phosphorylation (Fig. 2e, f) and nuclear translocation (Fig. 2e) induced by Kit-Fc, validating that the c-Kit ECD induces CREB S133 phosphorylation through direct interaction with mKitL.

### mKitL signaling activates the Akt pathway

**Fig. 3:**
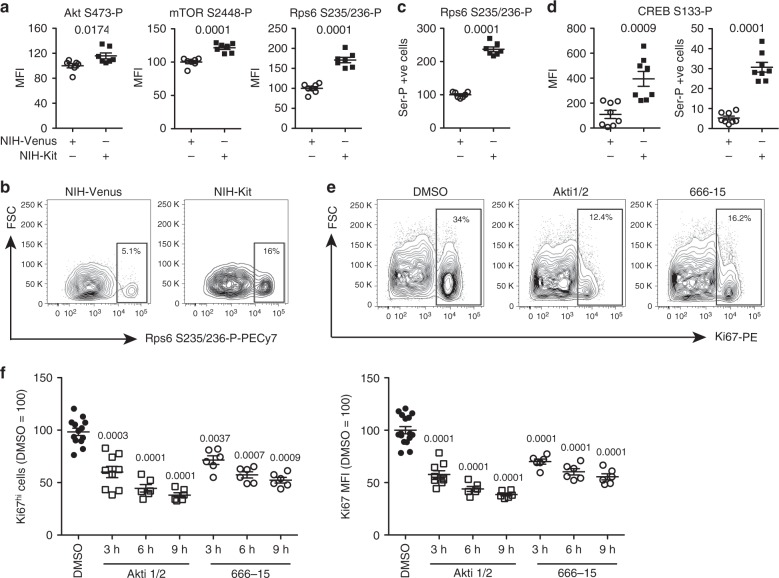
Activation of the Akt/mTOR pathway by reverse mKitL signaling. **a** Intracellular flow cytometry analysis of Akt S473, mTOR S2448, and Rps6 S235/S236 phosphorylation in NIH3T3 cells co-cultured with NIH-Venus or NIH-Kit cells as indicated (*N* = 7, two experiments). Values are normalized MFI (NIH-Venus mean = 100). Bars show mean ± s.e.m. *P*-value is shown (Student’s *t*-test). **b** Representative intracellular flow cytometry plots measuring Rps6 S235/S236 phosphorylation in NIH3T3 cells co-cultured with NIH-Venus or NIH-Kit cells, as indicated. Gating shows cells with high phospho-Rps6 levels. Data are representative of seven experiments. **c** Percentage of phospho-Rps6 positive cells in NIH3T3 cells co-cultured with NIH-Venus or NIH-Kit cells in **b** (*N* = 7, two experiments). Bars show mean percentage of S235/S236 positive cells ± s.e.m normalized to the average value for NIH-Venus ( = 100). *P*-value is shown (Student’s *t*-test). **d** CREB phopho-Ser133 MFI (left) and percentage CREB phopho-Ser133 positive cells (right) in NIH3T3 cells co-cultured with NIH-Venus or NIH-Kit cells, as indicated (*N* = 8, two experiments). Bars show mean ± s.e.m. *P-*value is shown (Student’s *t*-test). **e** Representative intracellular flow cytometry plots measuring Ki67 in NIH3T3 cells co-cultured with NIH-Kit cells and treated for 9 h with DMSO, Akt inhibitor (Akti1/2) or CREB inhibitor (666–15), as indicated. Gating shows the Ki67^high^ population. **f** Percentage of Ki67^high^ cells (left) and Ki67 MFI (right) in NIH3T3 cells co-cultured with NIH-Kit cells and treated for 3, 6, or 9 h (as indicated) with DMSO (*n* = 13, two experiments), Akt inhibitor (Akti1/2), or CREB inhibitor (666–15), as indicated (*N* = 6, two experiments, except the Akti1/2 3 h time point where *N* = 9, two experiments). Bars show mean ± s.e.m. *P-*value is shown (Student’s *t*-test)

Next, to globally identify signaling pathways activated downstream of KitL we performed phospho-proteome analysis of Kit-Fc and IgG-Fc treated NIH3T3 cells after 10 min of incubation, as this time point showed optimal induction of CREB and Rps6 phosphorylation after Kit-Fc incubation (Fig. 2a). A total of 427 proteins showed significantly altered phosphorylation (*P* < 0.05, *t*-test) in Kit-Fc-treated cells (Supplementary Data 1). Pathway analysis of the mKitL-regulated phospho-proteome showed extensive perturbation of mTOR signaling, eIF2 signaling and eIF4/p70S6-kinase signaling (Supplementary Data 2), indicating activation of the Akt/mTOR pathway^12^, and consistent with the observed induction of Rps6 S235/S236 phosphorylation after mKitL activation (Fig. 2b). Examination of specific phosphorylation states in NIH3T3 cells using intracellular flow cytometry confirmed increased Akt S473, mTOR S2448, and Rps6 S235/S236 phosphorylation (Fig. 3a–c), as well as increased S133 phosphorylation of CREB (Fig. 3d), after NIH-Kit co-culture. To determine the role of Akt and CREB in the proliferative response to mKitL activation NIH3T3 cells were co-cultured with NIH-Kit or NIH-Venus cells in the presence or absence of inhibitors of CREB or Akt. The previously observed proliferative response, as measured by Ki67 upregulation, was blocked by inhibition of either Akt or CREB (Fig. 3e, f). We therefore conclude that binding of the c-Kit ECD to mKitL leads to the activation of Akt and its downstream effectors mTOR and CREB in mKitL expressing cells, promoting their proliferation.

### CAML interacts with the mKitL ICD

**Fig. 4:**
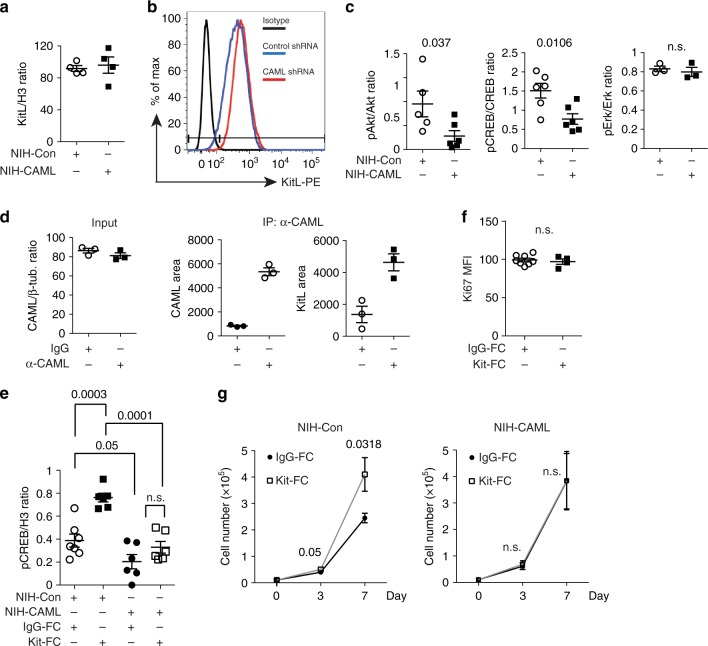
CAML is required for mKitL signal transduction and proliferation induction. **a** Expression of mKitL in NIH3T3 cells transduced with lentivirus expressing CAML shRNA (NIH-CAML) and Control shRNA (NIH-Con), as indicated. Protein levels were quantified by western blotting, and mKitL normalized to Histone H3 (*N* = 4, three experiments). Bars show mean ± s.e.m. *P*-value is shown (Student’s *t*-test). **b** Flow cytometry analysis of surface mKitL expression in cells from **a**, using an anti-KitL antibody or corresponding isotype control (Isotype). Data are representative of two experiments. **c** Quantification of phospho-Akt, -CREB and –Erk in NIH-CAML and NIH-Con cells gown in medium with 2% FBS. Protein levels were quantified by western blotting, and phospho-protein levels normalized to the corresponding total protein (*N* = 6, three experiments). Bars show mean ± s.e.m. *P*-value is shown (Student’s *t*-test); n.s. not significant. **d** Quantification of CAML input protein (left), and CAML (centre) and KitL protein immuno-precipitated by control IgG (IgG) and anti-CAML antibody (α-CAML) as indicated. Proteins were quantified by western blotting (*N* = 3). CAML input was normalized to β-tubulin. Bars show mean ± s.e.m. *P*-value is shown (Student’s *t*-test). **e** Quantification of CREB phopho-Ser133 in NIH-CAML and NIH-Con cells, incubated in 0.5% FBS medium for 24 h and stimulated with 200 ng/ml IgG-Fc or Kit-Fc for 8 h, as indicated (*N* = 6 in two experiments, except IgG-Fc-treated NIH-Con (*N* = 7, two experiments)). *P*-values are shown (Student’s *t*-test). **f** MFI of Ki67 expression in NIH-CAML cells grown in 2% FBS medium and stimulated with 200 ng/ml IgG-Fc or Kit-Fc for 8 h, measured by intracellular flow cytometry (*N* = 5, two experiments). Bars show mean ± s.e.m. *P*-value is shown (Student’s *t*-test); n.s. not significant. **g** Proliferation curve of NIH-Con (left) and NIH-CAML cells (right) grown in 2% FBS medium with 200 ng/ml IgG-Fc or Kit-Fc. Medium was changed every 48 h. Cells were counted at 0, 3, and 7 days (*N* = 6 for each time point, two experiments). Bars show mean ± s.e.m. *P*-values are shown (Student’s *t*-test); n.s. not significant

To further address the mechanism by which KitL transduces signaling we used the KitL intracellular domain (ICD) as bait in a yeast 2-hybrid screen, and identified several putative KitL-ICD interacting proteins (Supplementary Table 1). Of these calcium-modulating cyclophilin ligand (CAML) was phosphorylated upon mKitL activation (Supplementary Data 1), and CAML was previously shown to participate in TCR signaling^13^. Knockdown of CAML in NIH3T3 cells (Supplementary Fig 3) did not affect mKitL expression (Fig. 4a) or cell surface presentation (Fig. 4b). However, upon CAML knockdown Akt S473 and CREB S133 phosphorylation was decreased, whereas the Erk activation state was unaffected (Fig. 4c). Co-immunoprecipitation of CAML and mKitL confirmed their interaction in NIH3T3 cells (Fig. 4d, Supplementary Fig 4). Consistent with CAML-dependent activation of Akt and CREB mediating the proliferative response to mKitL activation, we observed that CREB activation after Kit-Fc stimulation was abolished by CAML knockdown (Fig. 4e), as was the proliferative response to mKitL activation as measured by Ki67 expression (Fig. 4f) and cell proliferation (Fig. 4g). CAML is therefore required for induction of signal transduction and cell proliferation upon activation of mKitL signaling.

### mKitL signaling promotes thymic VEC proliferation

**Fig. 5:**
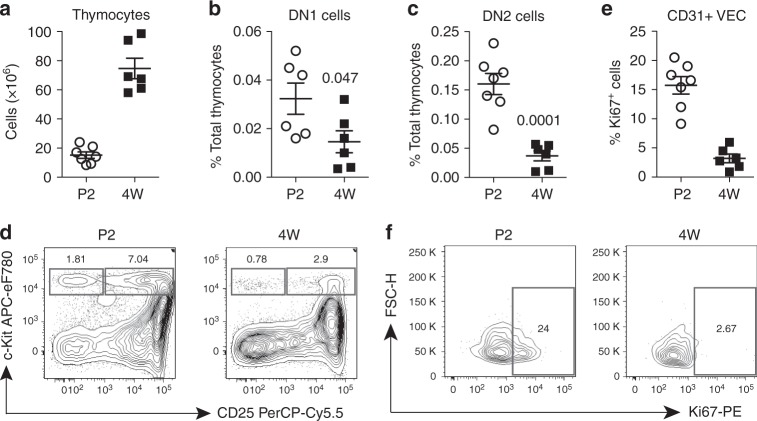
Thymic VEC proliferation is developmentally coordinated with ETP prevalence. **a** Total thymic cells from postnatal day 2 (P2) (*N* = 7, two experiments) and 4-week-old mice (4 W) (*N* = 6, two experiments) (cellularity from 1 lobe). Bars show mean ± s.e.m. **b** Percentage of phenotypically defined DN1 thymic progenitor cells (Dapi-Lineage-CD4-CD8-CD25-c-Kit+) from postnatal day 2 (P2) (*N* = 7, two experiments) and 4-week-old (4 W) mice (*N* = 6, two experiments). Bars show mean ± s.e.m. *P*-value is shown (Student’s *t*-test). **c** Percentage of phenotypically defined DN2 thymic progenitor cells (Dapi-Lineage-CD4-CD8-CD25+c-Kit+) from postnatal day 2 (P2) (*N* = 7, two experiments) and 4 weeks old (4 W) mice (*N* = 6, two experiments). Bars show mean ± s.e.m. *P*-value is shown (Student’s *t*-test). **d** DN1 and DN2 thymic progenitor cells from postnatal day 2 (P2) and 4-week-old mice (4 W). Plots are representative of two experiments. **e** Percentage of phenotypically defined Ki67+VECs (CD45-Ter119-EpCAM-CD31+) from postnatal day 2 (P2) and 4-week-old mice (4 W). Bars show mean ± s.e.m. **f** Ki67 expression in perinatal (P2) and 4-week-old mice (4 W) thymic VECs. Plots are representative of two experiments

**Fig. 6:**
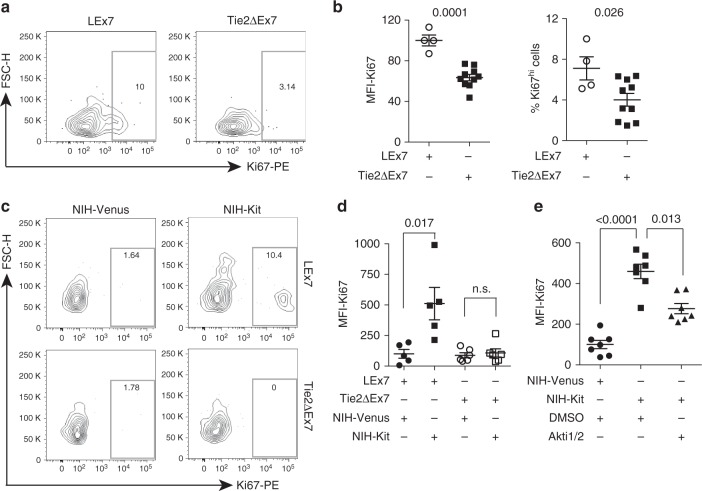
mKitL induces thymic VEC proliferation in vitro and in vivo. **a** Ki67 expression in perinatal (P2–P5) thymic VECs from LEx7 and Tie2∆Ex7 mice, as indicated, measured by intracellular flow cytometry. Plots are representative of two experiments. **b** MFI of Ki67 expression (left) and percentage of Ki67^hi^ cells (right) in perinatal thymic VECs from LEx7 and Tie2∆Ex7 mice from **a** (LEx7: *N* = 4, Tie2∆Ex7: *N* = 10 in two experiments). Bars show mean ± s.e.m. *P*-values are shown (Student’s *t*-test). **c** Ki67 expression in thymic VECs from LEx7 (left) and Tie2∆Ex7 mice (right), co-cultured with NIH-Venus or NIH-Kit cells as indicated and measured by intracellular flow cytometry. Plots are representative of two experiments. **d** MFI of Ki67 expression in thymic VECs from LEx7 (*N* = 5, two experiments) and Tie2∆Ex7 mice (*N* = 6, two experiments), co-cultured with NIH-Venus or NIH-Kit cells as indicated and measured by intracellular flow cytometry. Bars show mean ± s.e.m. *P*-values are shown (Student’s *t*-test). **e** MFI of Ki67 expression in thymic VECs, co-cultured with NIH-Venus (*N* = 7, two experiments), or NIH-Kit cells in the presence (*N* = 7, two experiments) or absence (*N* = 7, two experiments) of Akti1/2 for 6 h, measured by intracellular flow cytometry. Bars show mean ± s.e.m. *P*-values are shown (Student’s *t*-test)

In the thymus immigrating c-Kit+ ETPs initially remain in contact with cortical VECs (Supplementary Fig 5a), and VEC-associated mKitL supports their survival^5^. The frequency of c-Kit+ thymocytes is higher in perinatal, expanding thymus compared to fully expanded 4-week-old thymi (Fig. 5a–d; Supplementary Fig 5b; Supplementary Fig 6a), as is the proliferation of thymic VECs (Fig. 5e, f; Supplementary Fig 6a). We therefore tested if c-Kit expressed on thymocytes contribute to VEC proliferation during thymus expansion by analyzing mice, in which mKitL expression had been genetically ablated specifically in VEC. This was done by crossing mice with a *loxP*-flanked *Kitl* exon 7 (*Kitl*^LEx7^ allele), which encodes the trans-membrane domain of mKitL, to the VEC-specific *Tie2-Cre* driver, generating Tie2∆Ex7 mice (*Kitl*^LEx7/LEx7^; *Tie2-Cre*^tg/+^ genotype)^5^. Analysis of perinatal thymi from Tie2∆Ex7 and control mice (*Kitl*^LEx7/LEx7^ genotype; LEx7 mice) showed a significant reduction of Ki67-expressing, proliferating VEC upon VEC-specific mKitL depletion (Fig. 6a, b; Supplementary Fig 6a). To determine whether this effect was c-Kit-mediated, thymic VECs from LEx7 and Tie2∆Ex7 mice were co-cultured with NIH-Venus or NIH-Kit cells. Proliferation of control VECs was higher in NIH-Kit, compared to NIH-Venus, co-culture, and this effect was absent in the case of Tie2∆Ex7 VECs (Fig. 6c, d; Supplementary Fig 6b, c), demonstrating dependence of proliferation of thymic VECs on both c-Kit and mKitL. Finally, as observed for NIH3T3 cells, Akt inhibition led to a significant decrease in c-Kit-induced thymic VEC proliferation (Fig. 6e). We were not able to detect c-Kit expression on thymic VECs in vivo (Supplementary Fig 5a,b), nor did we observe any effect of Imatinib on VEC proliferation in NIH-Kit co-culture (Supplementary Fig 5c–e), indicating that also for VECs c-Kit activation is not required for the proliferative response elicited through mKitL.

## Discussion

Evidence that niche cells can depend on the stem- or progenitor cells they support has been obtained from the *Drosophila* ovary, where Cap niche cells maintain germline stem cells (GSCs) through BMP signaling^14^. Conversely, Delta ligand expressed on GSCs maintains Cap cells via Notch signaling. In the absence of Notch signaling, Cap cells, and subsequently GSCs, are lost^15^. We here show that thymic VEC proliferation is regulated by c-Kit-mediated activation of mKitL signaling, and that this pathway is rate limiting for VEC proliferation during postnatal thymus expansion. In conjunction with the finding that mKitL expression on cortical thymic VECs is required for the survival of c-Kit-expressing thymocyte progenitors^5^, this indicates mutual dependence of thymic progenitors and their niche cells. Importantly, crosstalk between mKitL+ thymic VECs and c-Kit+ thymocytes occurs simultaneously via a single molecular complex and requires cell–cell contact, enabling strict spatial control and selectivity of signaling.

Bi-directional signaling has previously been observed for Eph-ephrin complexes, with their most important roles being in patterning of the vasculature and central nervous system^16,17^. Ephrin expression in neurons has been shown to inhibit proliferation of the neuronal stem and progenitor cells that generate them^18^, and reciprocal regulation of EphB2/B3 and EphrinB1/B2 by Wnt signaling in the intestine restricts progenitor cell proliferation to the crypt^19^, both examples of negative feedback signaling that contributes to tissue homeostasis.

In contrast, mKitL–c-Kit bi-directional signaling supports the simultaneous expansion of interacting mKitL and c-Kit-expressing cells, here exemplified by c-Kit+ thymocyte progenitors and their supporting mKitL+ vascular endothelial stromal cells. Such a bi-directional signal would be suitable to drive coordinated cellular expansion in developing and regenerating tissues. Relevant to this, local KitL signaling has been proposed to sustain hematopoietic stem cells (HSCs) not only under steady state conditions^20^, but also during the expansion of facultative HSCs niches in the spleen during extramedullary hematopoiesis^21^. Furthermore, reprogramming of stromal cells by cancer cells to enhance their ability to support tumor progression has been observed in both hematopoietic^22^ and solid cancers^23,24^, and c-Kit is widely expressed in malignant cancers^1^, and supports the maintenance of cancer stem cells in several tumor types^25^, including lung^26^ and ovarian cancers^27^. The molecular mechanisms that mediate stromal adaptation include *PTEN* downregulation^23^ and *Hmga2* overexpression^28^, both of which activate Akt signaling^29–31^, and upregulation of HSF1^24^, a transcription factor activated by mTOR-mediated phosphorylation^32^. Bi-directional mKitL–c-Kit signaling may therefore contribute to the interaction between cancer cells (including cancer stem cells) and stromal cells that generates reactive stroma through Akt/mTOR pathway activation. Combined with the importance of c-Kit–KitL interaction for the development and maintenance of multiple tissues^1^ bi-directional c-Kit–mKitL signaling may therefore be important for both normal and malignant cell–cell interactions.

## Methods

### Cell lines

NIH3T3 cells (ATCC CRL-6442) were cultured in IMDM with heat inactivated foetal calf serum and penicillin/streptomycin (Gibco) at 37 °C and 5% CO_2_. Standard culture was in 10% FCS with serum percentage adjusted is indicated according to the type of experiment performed.

For CAML knockdown 5 × 10^5^ NIH3T3 were seeded in 6-well plates and infected overnight with hairpin-expressing lentivirus. Medium was replaced and cells kept or 2 additional days before cell passage and puromycin selection (1 μg/ml). CAML expression was analysed by western blot and cells with optimal CAML knockdown (NIH-CAML) and control infected cells (NIH-Con) expanded under puromycin selection. Information regarding the lentiviral vector and CAML shRNA sequences can be found in Supplementary Table 3.

NIH-Venus and NIH-Kit were generated by infection of NIH3T3 cells with empty pRRL-Venus and pRRL-Venus-c-Kit, respectively, the latter expressing full-length murine c-Kit. Venus expressing cells were isolated by FACS. Cultures were regularly monitored for GFP expression.

For co-culture experiments NIH3T3 cells were cultured in the presence of 2% FBS with an equal number of NIH-Venus or NIH-Kit cells for 24 h. For drug treatment drugs were added at this time point, and the culture continued for the indicated time. The ATP binding domain point mutation K623M^11^ of the c-Kit coding sequence has been performed by NEB QC mutagenesis. NIH-Kit-K623M were generated by infection of NIH3T3 cells with pRRL-Venus-c-Kit-K623M. NIH-Kit and NIH-Kit-K623M were subsequently FACS sorted based on c-Kit expression in order to achieve the same c-Kit expression level for the two cell lines.

### Kit-Fc stimulation

For phospho-protein readout 3 × 10^5^ NIH3T3 cells (or NIH-CAML and NIH-Con) were plated in the presence of 0.5% FBS for 24 h. Medium was 2 additional hours prior to addition of 200 ng/ml IgG-Fc (Sino Biological 10702-HNAH) or Kit-Fc chimera (Sino Biological 50530-M02H-50). After the indicated time cells were washed with PBS and protein lysates prepared for western blot or intracellular flow cytometry.

For phopho-flow cytometry were then collected following trypsinization and re-suspended in 50 μl of cytofix/cytoperm (BD) and left for 30 min in ice before washing in BD wash/perm buffer. The antibodies (anti-Ki67-PE; anti-phospho-Akt-APC; anti-phospho-mTOR-eF450; anti-phospho-Rps6-PECy7; anti-phospho-CREB and goat anti-rabbit AlexaFluor488) were incubated overnight at 4 °C. The cells were then washed in BD wash/perm buffer and FACS analysed.

For Ki67 and BrdU readout NIH3T3 cells were grown in the presence of 2% FBS for 24 h and the medium changed before applying 200 ng/ml IgG-Fc or Kit-Fc for 8 or 24 h prior to analysis, as indicated. For BrdU incorporation a BrdU Flow kit (Becton Dickinson) was used according the manufacturer’s instructions. BrdU was added 2 h prior to analysis. Cells were then collected by trypsinization and re-suspended in 50 μl of cytofix/cytoperm (Becton Dickinson) and left for 30 min in ice before washing in BD wash/perm buffer. Cells were then centrifuged at 500×*g* for 5 min and the pellet re-suspended in DNaseI solution for 1 h at 37 °C. After washing cells were incubated with anti-BrdU-eF450 antibody overnight at 4 °C, washed in BD wash/perm buffer and analysed by flow cytometry.

For ELISA assays protein lysates were prepared according to the manufacturer’s instructions (abcam), and CREB phospho-S133 (ab176659), Erk1 phospho-T202/Y204 + Erk2 phospho-T185/pY187 (ab126445), and p38α phospho-T180/pY182 (ab126453) measured along with the total of the same proteins using the indicated ELISA kits. The readout was carried out by using a CLARIOstar® ELISA reader (BMG LABTECH).

For antibody neutralization NIH3T3 cells were grown in the presence of 0.5% FBS for 24 h. At this time medium was changed and goat-IgG (2 μg) or goat-anti-mouse-SCF (2 μg) was added to the medium for 1 h before stimulating with IgG-Fc or Kit-Fc for 10 min. The cells were then washed in cold PBS and protein lysates prepared.

### Cell proliferation assay

For measurement of cell proliferation 1 × 10^4^ NIH-Con or NIH-CAML cells were plated in 10 cm dishes and cultured in IMDM/2% heat inactivated serum/ PS for 7 days. IgG-Fc or Kit-Fc stimulation was applied every other day (starting from day 0) and cells counted at days 3 and 7.

### Immunofluorescence

NIH3T3 cells were plated onto Millicell EZ 8-well Glass slides and cultured for 24 h in 0.5% FBS. Medium was changed and the cells incubated for 2 additional hours. IgG-Fc or Kit-Fc was added for 10 min before washing the cells and fixing in acetone for 5 min. The slides were then air dried and re-hydrated for 30 min in PBS at room temperature and blocked in 5% BSA in PBS for 10 min at room temperature. Anti CREB phospho-Ser133 primary antibody was diluted in PBS and slides stained for 2 h and then washed several times in PBS before adding the secondary antibody for 45′ (goat anti-rabbit AlexaFluor488) at room temperature. Slides were then counterstained with DAPI (0.2 μg/ml) and mounted with DXP mounting medium (Fisher Scientific). Images were acquired with a Zeiss 780 confocal inverted microscope and processed using Fiji software.

### Thymic section preparation and imaging

Freshly isolated thymi were washed in PBS and included in OCT for snap freezing. The OCT blocks were sliced at 7 μm thickness and sections stored at −80 °C. The sections were dried at room temperature for 5 min before fixing in acetone for 5 min at 4 °C and left at room temperature for 5 min to ensure acetone evaporation. The sections were then re-hydrated for 30 min in PBS at room temperature and blocked in 5% BSA in PBS for 10 min at room temperature. Fluorochrome-conjugated or unconjugated primary antibodies were diluted in PBS and sections stained for 1 h and then washed several times in PBS before adding the secondary antibody for 45 min at room temperature. Sections were then counterstained with DAPI (0.2 μg ml^−1^) and mounted with DXP mounting medium (Fisher Scientific). Images were acquired with a Zeiss 780 confocal inverted microscope and processed using Fiji software.

### Protein lysates preparation and western blot

NIH3T3 were lysed in RIPA buffer (150 mM sodium chloride, 1.0% NP-40 or Triton X-100, 0.5% sodium deoxycholate, 0.1% sodium dodecyl sulfate (SDS), 50 mM Tris-HCl, pH 8.0) supplied with cOmplete Protease Inhibitor Cocktail (Roche) and PhosSTOP – Phosphatase Inhibitor Cocktail Tablets (Roche). Cells were kept for 20 min in ice and an aliquot set aside to determine the protein concentration with Qubit protein reagent. 6X protein loading buffer (30% glycerol, 0.5 M Tris-HCl, pH 8.0, 6 mM EDTA, 10% SDS, 10% β-mercaptoethanol, 0.01% bromophenol blue) was then added and samples sonicated for 5 min in an ultrasonic bath (Qsonica; Amplitude 50; 30 s ON, 30 s OFF) before boiling for 5 min at 100 °C. Twenty micrograms of total protein lysate was loaded onto NuPAGE 4–12% Bis-Tris Protein Gels (Thermo Fisher). Protein transfer onto PVDF membrane was performed using Trans-Blot SD Semi-Dry Transfer Cell. Membranes were washed in TBS/0.001% Tween-20. Primary antibodies were diluted in 5% milk in TBS/0.001% Tween-20 and incubated overnight at 4 °C on a rocking platform. Secondary antibodies were diluted in 5% milk in TBS/0.001% Tween-20 and incubated for 1 h at RT on a rocking platform. Signal was developed by using ECL western blot substrate (Pierce) and acquired onto CL-XPosure™ Film (Thermo Scientific).

### Co-immunoprecipitation

NIH3T3 cells from a 10 cm dish were lysed in RIPA buffer supplied with cOmplete Protease Inhibitor Cocktail by gently scraping, and lysates clarified by centrifugation at 13,000 g for 10 min at 4 °C. The supernatant was collected and an aliquot stored as input. The rest of the material was incubated with polyclonal anti-CAML antibody (Santa Cruz Biotechnology, San Diego, CA; Supplementary Table 2) for 3 h at 4 °C on a rotating wheel. Forty microliters of Dynabeads protein-G, washed twice in 1 ml of RIPA buffer with protease inhibitors on the rotating wheel at 4 °C for 10 min, was added and incubation continued for additional 45 min, followed by washing. The protein-antibody-beads complexes were then isolated on a magnetic rack and re-suspended in RIPA buffer with protease inhibitors and protein loading buffer. The eluted material was used to load two replica gels that were probed with anti-CAML and the anti-mKitl antibody, respectively. Primary antibody was detected with protein A-HRP (CST).

### Co-culture of primary thymic cells

Thymic stromal cells were prepared from 4 to 6-week-old LEx7 and Tie2∆Ex7 mice. Dissected thymi were finely chopped and the obtained fragments digested in a mix of Collagenases I and 3 (Worthington; 3 mg ml^−1^), dispase II (Roche; 7 mg ml^−1^), DNaseI (Invitrogen; 2 U ml^−1^) at 37 °C with gentle agitation for 20 min. The dissociated cells were centrifuged at 300×*g* for 5 min and re-suspended in culture medium. The total thymic cell population was divided in two aliquots and seeded onto 2 × 10^6^ NIH-Venus and NIH-Kit cells, respectively, in 10 cm tissue culture dishes. Cells were co-cultured for 24 h before collecting for cell surface staining followed by intracellular Ki67 staining. For drug treatment total thymic cells were seeded in 3 aliquots onto 1 × 10^6^ NIH-Venus or NIH-Kit cells in 6 cm tissue culture dishes. Drug or vehicle (DMSO) was added for additional 6 h. Cells were cultured in IMDM with 10% FBS throughout. Mice were maintained in accordance with protocols ethically approved by the Oxford University Clinical Medicine Animal Welfare and Ethics Review Board and the UK Home Office.

### Flow cytometry

Cell lines were collected by scraping or light trypsinization and re-suspended in PBS, 5% FBS. The antibodies used are listed in Supplementary Table 2. Cells were stained on ice for 20 min in the dark. Data were acquired using LSR Fortessa X-20 (BD Biosciences) and analysed using FlowJo software (TreeStar). Thymocyte and thymic VECs were quantified by flow cytometry using the antibodies described in Supplementary Table 4.

### Phospho-proteomic analysis

Cells were stimulated with Kit-Fc or IgG-Fc in 3 up-scaled (3×) replicates each as described above. The obtained lysates were reduced with 200 mM TCEP for 1 h at 55 °C and alkylated with 375 mM iodoacetamide for 30 min at room temperature in the dark. Proteins were precipitated with chloroform and methanol^33^ twice to remove detergents, before re-suspension in 50 mM TEAB and digest with 30 µg of trypsin per sample overnight at 37 °C. Samples were dried and re-suspended in 200 µl 50 mM TEAB for TMT labeling. Peptide concentrations were quantified using the Pierce Quantitative Colorimetric peptide assay following manufacturer instructions (Thermo Fisher). Equal amounts of each sample (465 μg) were labeled with TMT6 reagents (Thermo Fisher) for 1 h according to the manufacturer’s instructions before the labeling reaction was quenched with 5% hydroxylamine. The six samples were pooled and desalted using a C18 Sep-Pak cartridges (Waters): The cartridge was conditioned with 5 ml buffer B (0.1% TFA in 65% CH_3_CN), equilibrated with 10 ml of buffer A (0.1% TFA in 2% CH_3_CN). The tryptic peptides were added to bind to the cartridge and then washed with 10 ml buffer A. The peptides were eluted in 1.2 ml of buffer B and dried in a vacuum concentrator. Peptides were solubilised in 120 µl of 80% CH_3_CN/0.1% TFA solution.

Phospho-peptide enrichment was performed based on a refinement^34^ of the 2-step enrichment procedure published by McNulty et al.^35^. Fractionation was performed on a Dionex HPLC using a TSKgel Amide-80 column (5 µm, 4.6 mm ID × 25 cm, TOSOH) for hydrophilic interaction. The peptides were eluted at a 500 µl/minute flow rate using the following gradient: 10 min with 80% buffer B (98% CH_3_CN with 0.1% TFA), then from 10 to 55 min 80 to 60% of buffer B. The column was then washed for 10 min with 98% buffer A (98% water with 0.1% TFA) and the column was re-equilibrated for another 10 min with 80% buffer B. The collection was performed from 10 to 70 min each fraction was collected for 4 min. A total of 15 fractions were collected. Neighboring fractions were combined into 6 pools and 100 µl of PHOS-Select™ Iron affinity beads (Sigma) were added to each pool. The samples were incubated 1 h at 4 °C and filtered at 8200 g for 1 min over a 0.22 μm nylon Spin-X cartridge (Sigma) to remove un-phosphorylated peptides. The beads were successively washed with 500 µl of 250 mM of acetic acid with 30% CH_3_CN and 500 µl of water and centrifuged for 2 min at 2000 rpm after each wash. Phospho-peptides were eluted by incubation of the beads in 500 µl of 1% ammonium hydroxide for 10 min, followed by a centrifugation at 8200×*g* for 2 min. Samples were dried down and re-suspended in 10 µl of 1% CH_3_CN 0.1% TFA prior to mass spectrometry analysis.

### LC–MS/MS analysis

Samples were analysed in an Orbitrap fusion Lumos coupled to a UPLC ultimate 3000 RSLCnano System. The samples were loaded in 1% CH_3_CN 0.1% TFA buffer and eluted using a gradient of 2–35% CH_3_CN in 0.1% formic acid and 5% DMSO in 120 min at a flow rate of 250 nl/min.

The survey scan was acquired within a 380–1500 m/z window at a resolution of 120,000 and an AGC target of 2E5. Selected precursor ions were isolated in the quadrupole with a mass isolation window of 1.6Th and analysed in the Ion trap following CID fragmentation and detection in rapid mode. Peptide quantitation was based on synchronous precursor selection for the 10 most abundant MS2 fragments for a MS3 scan at a resolution of 60,000 in the Orbitrap detector with a normalized collision energy of 65% (HCD) and a scan range of 120–500 m/z. The total cycle time was fixed at 4 s with a dynamic precursor exclusion of 7 s.

### Proteomics data analysis

Raw files were imported into Proteome Discoverer (2.1) and analysed using the standard workflow for TMT-6plex and synchronous precursor selection: MS2 spectra were searched with SequestHT using 10 ppm as precursor and 0.5 Da as fragment error tolerance against the Uniprot/Swissprot database of *Mus musculus* (retrieved 17.07.2015). Variable modifications were methionine oxidation, phosphorylation on serine, threonine or tyrosine and N-terminal protein acetylation. Static modifications were cabamidomethylation on cysteine and TMT-6plex modification of the peptide N-terminus and lysine. Identifications were re-processed with Percolator to match a FDR of 1%. Quantitative data were analysed and visualized with Perseus^36^. Canonical Pathway Analysis was performed using Ingenuity Pathway Analysis (Qiagen).

### Yeast 2-hybrid screening

The murine KitL intracellular domain was cloned into GBT9 and used to screen a normalized human universal cDNA library (Clontech)^37^. Positive preys were sequenced and those known to be promiscuous based on previous screens, as well as those encoded only by 3′-UTR sequence, discarded. Hits are shown in Supplementary Table 1.

### Statistical analysis

Unpaired, two-tailed Student’s *t*-test was used throughout for data analysis

## Electronic supplementary material

Supplementary Data 2

Supplementary Information

Description of Additional Supplementary Files

Supplementary Data 1

## Data Availability

The mass spectrometry proteomics data have been deposited to the ProteomeXchange Consortium via the PRIDE partner repository [http://proteomecentral.proteomexchange.org] with the dataset identifier [PXD011061]. All other primary data are available from the authors upon request.
