# Supplementary material for: Bi-directional signaling by membrane-bound KitL induces proliferation and coordinates thymic endothelial cell and thymocyte expansion
Source: Nat Commun. 2018 Nov 8;9:4685. doi: 10.1038/s41467-018-07024-0 (PMC6224562; doi:10.1038/s41467-018-07024-0)
Supplement: Supplementary file 2 — Supplementary Information [file 41467_2018_7024_MOESM2_ESM.pdf]

Supplementary Information.

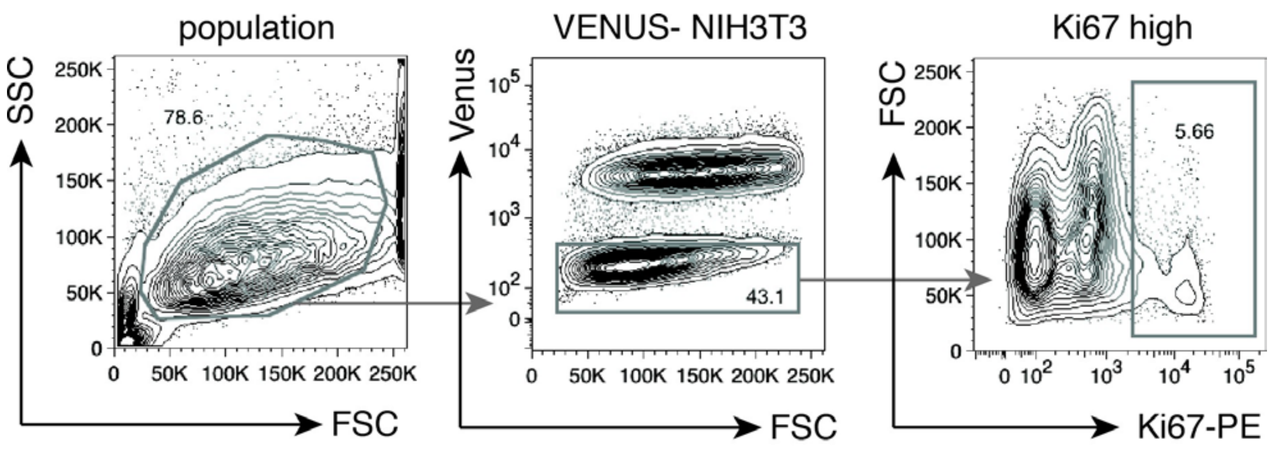

Supplementary Figure 1

Supplementary Figure 1: Gating strategy for NIH3T3 co-cultures.

Gating strategy used to identify un-transduced NIH3T3 cells (Venus–) in co-culture experiments with Venus-expressing NIH-Venus or NIH-Kit cells (Venus+) shown in Figure 1c,d,e and Figure 2a,b,c,d,e,f,g.

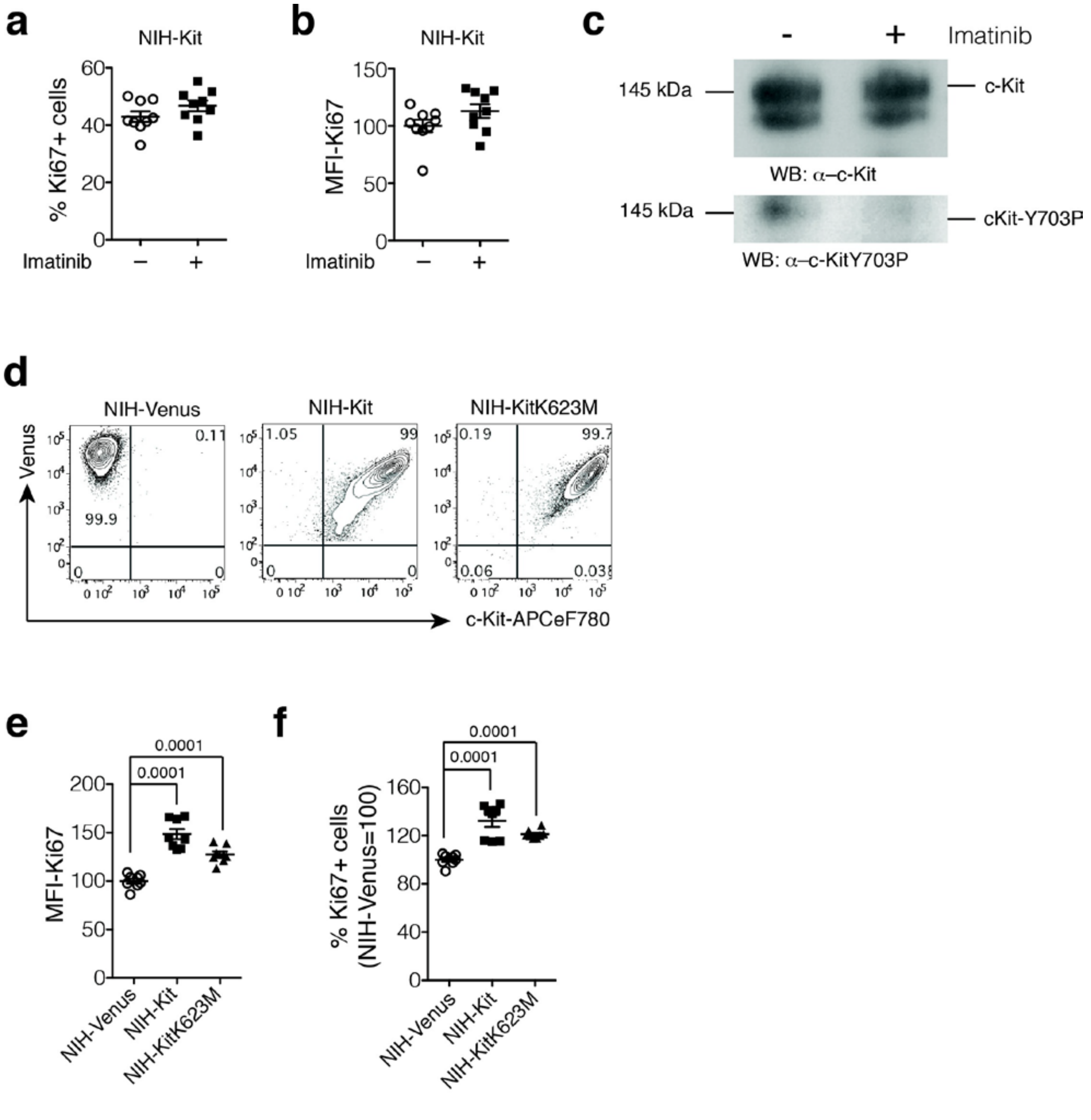

Supplementary Figure 2

<u>Supplementary Figure 2: Activation of c-Kit is dispensable for reverse KitL signaling in NIH3T3 cells.</u>

(a) Percentage of $Ki67^+$ cells NIH3T3 co-cultured with NIH-Kit cells and NIH-Kit cells in the presence of 10 μM Imatinib for 24 hours. (N=8 for both conditions, two experiments). Bars show mean±s.e.m. *P* values are shown (Student's t-test); n.s. = not significant.

(b) Mean fluorescence intensity (MFI) of Ki67 signal in samples from (a). Bars show mean±s.e.m. with NIH-Kit mean (- Imatinib) = 100. *P* values are shown (Student's t-test); n.s. = not significant.

(c) Western blot analysis of c-Kit (upper panel) and c-Kit-Y703P (bottom panel) from NIH-Kit treated with vehicle or Imatinib 10 μM Imatinib for 1 hour.

(d) Flow cytometry plots of pRRL-Venus–transduced (left plot; NIH-Venus) and pRRL–c-Kit–Venus (right plot; NIH-Kit) and pRRL–c-KitK623M–Venus transduced NIH3T3 cells stained with anti–c-Kit antibody. Data are representative of two experiments.

(e) Mean fluorescence intensity (MFI) of Ki67 signal in NIH3T3 co-cultured together with NIH-Venus or NIH-Kit or NIH-KitK623M detected by intracellular flow cytometry. Gating shows $Ki67^+$ cells (N=8 for all conditions, two experiments). Bars show mean±s.e.m. *P* value is shown (Student's t-test).

(f) Percentage of $Ki67^+$ NIH3T3 cells co-cultured together with NIH-Venus or NIH-Kit or NIH-KitK623M detected by intracellular flow cytometry. Gating shows $Ki67^+$ cells (N=8 for all conditions, two experiments). Bars show mean±s.e.m. with NIH-Venus mean = 100 *P* value is shown (Student's t-test).

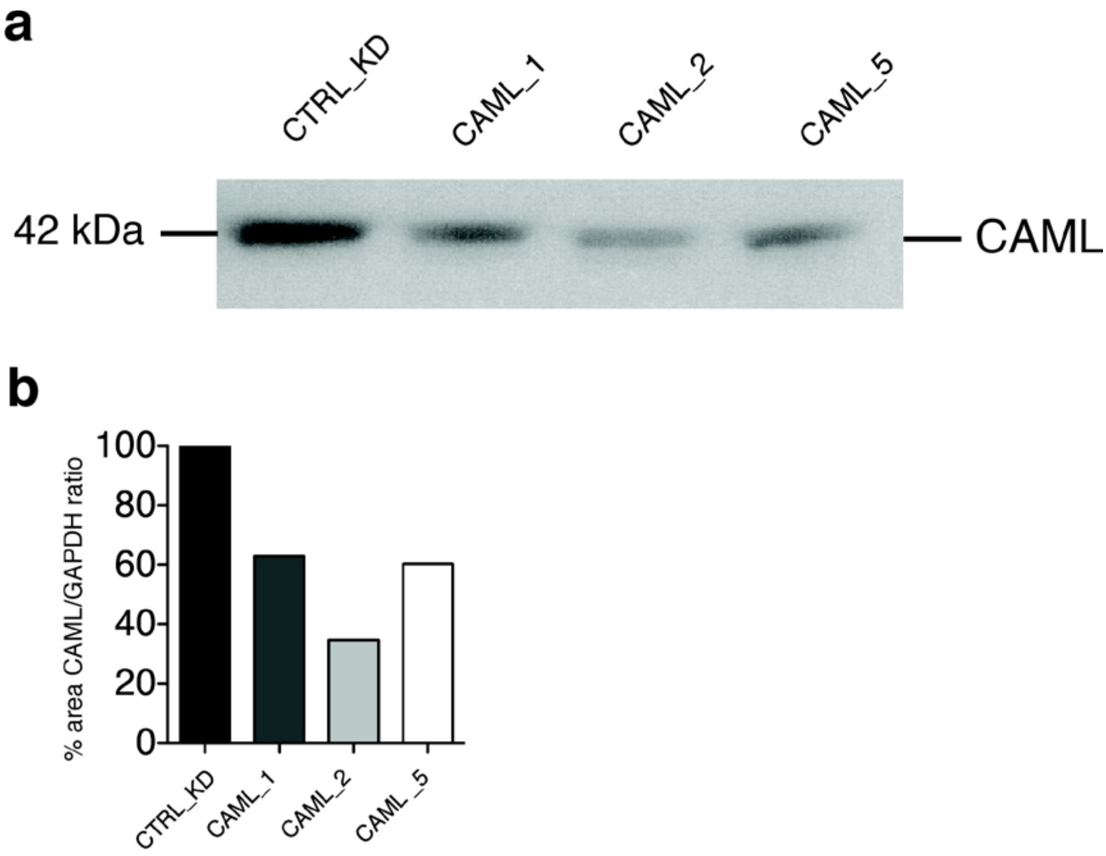

Supplementary Figure 3

Supplementary Figure 3: Measurement of CAML knockdown efficiency.

(a) CAML western blot analysis of NIH3T3 transduced with control and CAML lentiviral vectors.

(b) Quantification of CAML lentiviral knockdown. The graph represents the ratio between the areas of CAML and GAPDH western blot bands relative to control virus infected cells. Cells transduced with the CAML_2 shRNA were used for subsequent studies.

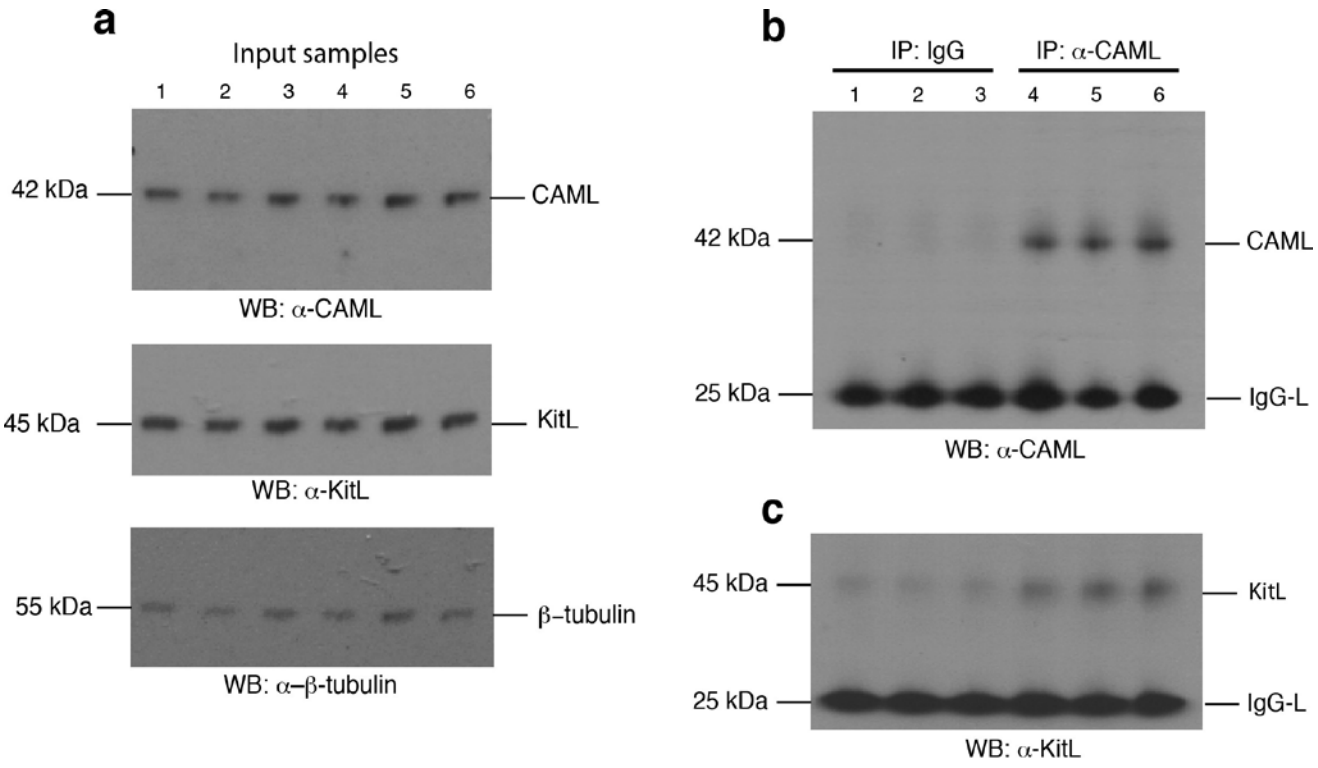

Supplementary Figure 4

Supplementary Figure 4: CAML-KitL co-immunoprecipitation.

(a) Western blot analysis of CAML (upper panel), KitL (central panel) and β-tubulin (bottom panel) input protein (N=3).

(b) Western blot analysis of CAML (upper panel) and KitL protein immuno-precipitated by control IgG (IP: IgG, N=3) and by anti-CAML antibody (IP: α-CAML, N=3) as indicated.

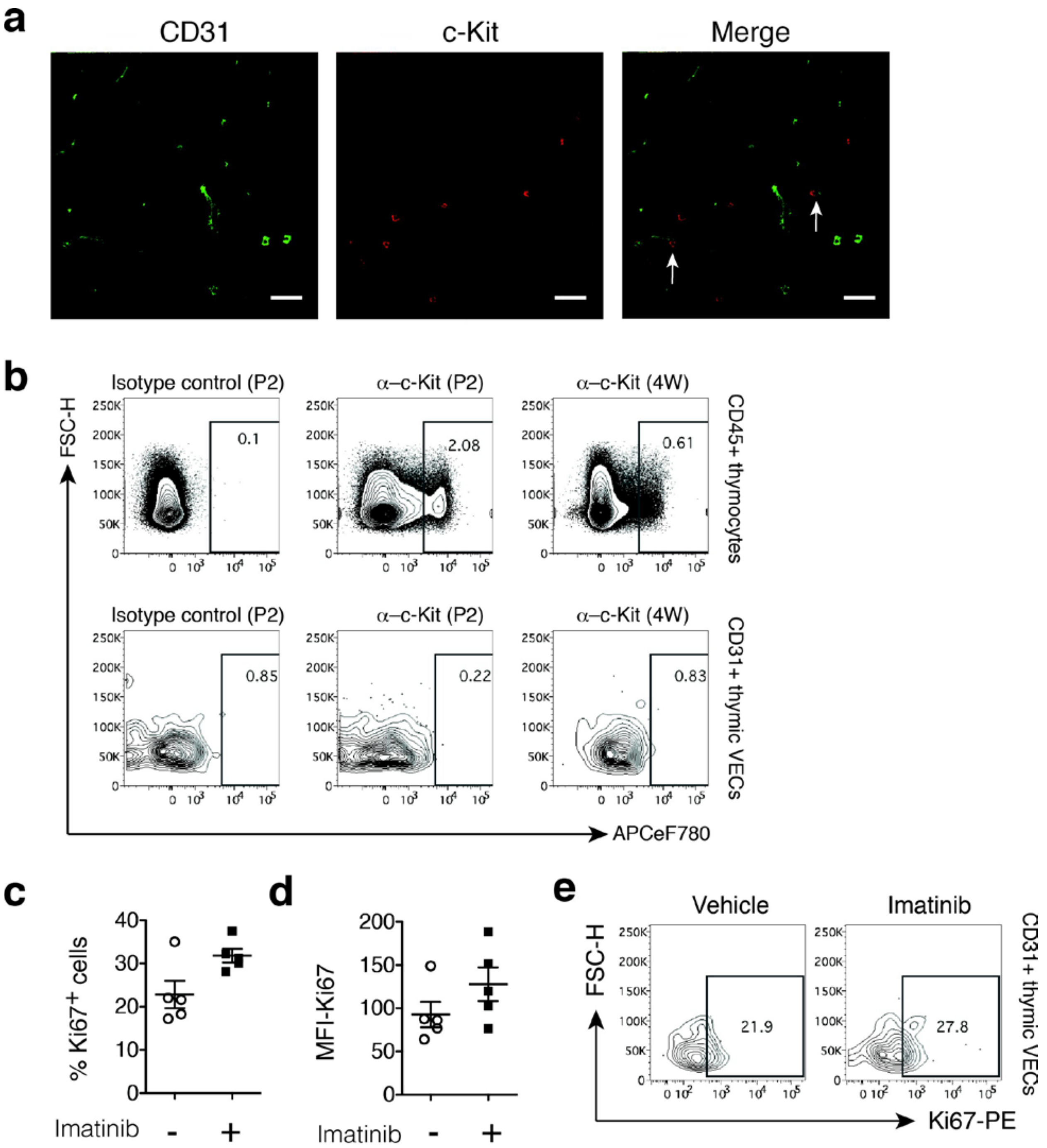

Supplementary Figure 5

Supplementary Figure 5: c-Kit expression on thymic CD45+ cells and CD31+ VECs.

(a) Representative immunofluorescence analysis of CD31 (green) and c-Kit (red) expression in 4-weeks old thymus. Scale bar: 100μm. Note the lack of c-Kit–CD31 co-localization.

(b) Expression of c-Kit on CD45+ thymocytes, gated as in Supplementary Fig 6, from postnatal day 2 (P2; middle panel) and 4 weeks old thymi (right panel). The isotype control staining from P2 thymus is shown for gating purposes (upper panels).

Expression of c-Kit on CD31+ thymic VECs, gated as in Supplementary Fig 6, from postnatal day 2 (P2; middle panel) and 4-weeks old thymi (right panel). The isotype control staining from P2 thymus is shown for gating purposes (lower panels).

(c) Percentage of Ki67$^+$ cells in in thymic VECs from 4-weeks old mice, co-cultured with NIH-Kit cells and NIH-Kit cells in the presence of 10 μM Imatinib for 24 hours. (N=5 for both conditions, two experiments). Bars show mean±s.e.m. *P* values were determined using Student's t-test and were not significant.

(d) Mean fluorescence intensity (MFI) of Ki67 signal in samples from (c). Bars show mean±s.e.m. with NIH-Kit mean (w/o Imatinib) = 100. *P* values were determined using Student's t-test and were not significant..

(e) Representative flow cytometric analysis of Ki67 expression in thymic VECs from (c). Plots are representative of two experiments.

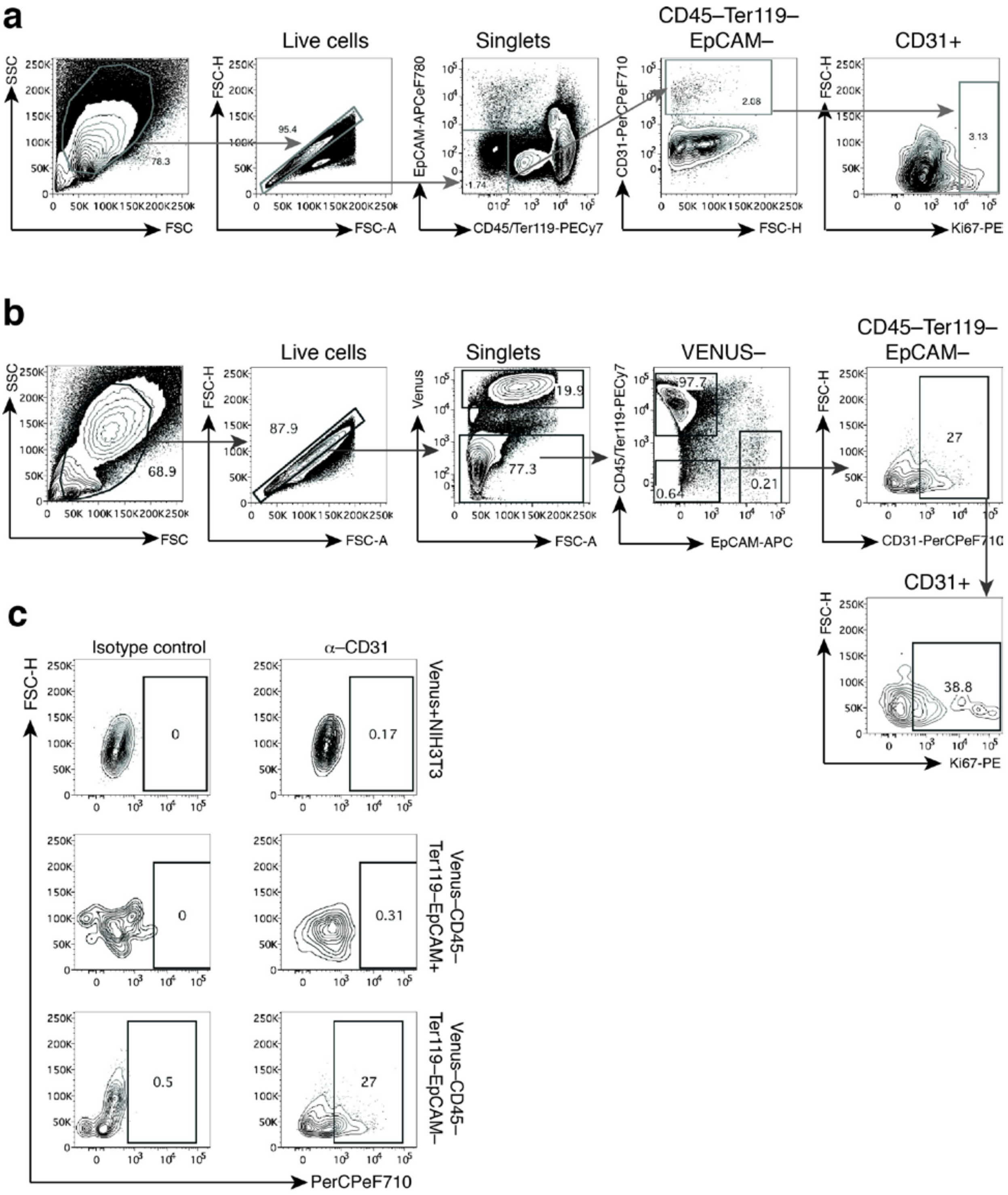

Supplementary Figure 6

Supplementary Figure 6: Gating strategy for thymic VEC identification.

(a) Gating strategy used to identify thymic VECs (CD45–Ter119–EpCAM–CD31+) and measurement of Ki67 expression *in vivo* shown in Figure 5e,f and Figure 6a,b.

(b) Gating strategy used to identify thymic VECs (Venus–CD45–Ter119–EpCAM–CD31+) and measurement of Ki67 expression in NIH3T3 co-culture experiment showed in Figure 6c,d,e) and Supplementary Figure 6c,d,e.

(c) Gating strategy of Venus+NIH3T3, Venus-CD45-Ter119-EpCAM+ and Venus-CD45-Ter119-EpCAM- to check for the specificity of the CD31 antibody against its specific isotype control (applies to Figure 5e,f and Figure 6a,b).

Supplementary Table 1: Positive hits in yeast two-hybrid screen using the KitL ICD as bait. In addition to the gene symbol of each positive hit the number of independent isolates, the cDNA start site and gene description is listed.

Prey gene symbol	Number of times isolated	Start in 5'UTR	Start in coding sequence	Number of bases of the 5'UTR that are part of the hybrid prey protein	Description
APOE	12	0	12	0	apolipoprotein E
GIMAP4	6	0	6	0	GTPase, IMAP family member 4
ASAP1-IT1	2	0	2	0	ASAP1-IT1 ASAP1 intronic transcript 1 (non-protein coding) [Homo sapiens]
POLG2	2	0	2	0	polymerase (DNA directed), gamma 2, accessory subunit
TTC1	2	0	2	0	tetratricopeptide repeat domain 1
XKRY	1	1	0	243	XK, Kell blood group complex subunit-related, Y-linked
XKRY2	1	1	0	243	XK, Kell blood group complex subunit-related, Y-linked 2
CAMLG	1	1	0	77	calcium modulating ligand
C2orf77	1	0	1	0	chromosome 2 open reading frame 77
C8orf71	1	0	1	0	chromosome 8 open reading frame 71
PPIA	1	0	1	0	peptidylprolyl isomerase A (cyclophilin A)
PAPOLA	1	0	1	0	poly(A) polymerase alpha

Supplementary Table 2: List of antibodies used, including their application, working concentration, manufacturer and, where relevant, clone number.

Antibodies used for the study				
**Antibody**	**Application**	**Working concentration/Volume**	**Manufacturer**	**clone**
Goat anti mouse SCF biotin	FACS, neutralization	5 μg/ml; 2 μg/ml	R&D systems	BAF455
c-Kit APCef780	FACS	0.25 μg/ml	eBioscience	2B8
Anti Human and Mouse Ki67 PE	FACS	1 in 25 dilution	Miltenyi Biotech	REA183
Anti BrdU eF50	FACS	2.5 μg/ml	eBioscience	BU20A
Phospho-AKT1 (Ser473) APC	FACS	1 in 20 dilution	CST	monoclonal antibody SDRNR
Phospho-mTOR (Ser2448) eFluor 450	FACS	1 in 20 dilution	CST	monoclonal antibody MRRBY
Phospho-S6 (Ser235, Ser236) PECy7	FACS	1 in 20 dilution	CST	monoclonal antibody cupk43k
Phospho-CREB (Ser133)	FACS, WB, IF	1 in 20 dilution; 1:500; 1:100	CST	87G3; Rabbit monoclonal
Normal Goat IgG Biotinylated Control	FACS, neutralization	5 μg/ml; 2 μg/ml	R&D systems	BAF108
CD45 PECy7	FACS	0.33 μg/ml	eBioscience	30-F11
Ter-119 PECy7	FACS	0.33 μg/ml	eBioscience	TER-119
CD31 PerCP eF710	FACS	0.8 μg/ml	eBioscience	390
EpCAM APC eFluor780	FACS	0.5 μg/ml	eBioscience	G8.8
Sptreptavidin PE	FACS	1 μg/ml	eBioscience	
polyclonal anti CAML (sc-13970)	WB, IP	0.4 μg/ml; 2 μg/ml	SCBT	discontinued?
Phospho-AKT1 (Ser473) Antibody	WB	2 μl/ml	CST	#9271; Rabbit polyclonal
AKT1 Antibody	WB	2 μl/ml	CST	2H10; Mouse monoclonal
Phospho-p44/42 MAP Kinase (ERK1/2) (Thr202/Tyr204)	WB	1 μl/ml	CST	D13.14.4E; Rabbit monoclonal
p44/42 MAP Kinase (ERK1/2)	WB	1 μl/ml	CST	L34F12; Mouse monoclonal
CREB1	WB	2 μl/ml	CST	86B10; Mouse monoclonal
Phospho-c-Kit/CD117 pTyr730 Antibody	WB	1 in 200 dilution	Thermo Scientific	polyclonal rabbit antibody
Human/Mouse CD117/c-kit Antibody	WB	0.1 μg/ml	R&D systems	AF1356 Polyclonal Goat IgG
CD3ε APC/Lineage cocktail ETPs staining	FACS	2 μg/ml	eBioscience	Rat monoclonal
Mac1 APC/Lineage cocktail ETPs staining	FACS	0.5 μg/ml	eBioscience	Rat monoclonal M1/70
TCRβ APC/Lineage cocktail ETPs staining	FACS	0.125 μg/ml	eBioscience	Rat monoclonal H57-597
Gr1 APC/Lineage cocktail ETPs staining	FACS	0.5 μg/ml	eBioscience	Rat monoclonal RB6-8C5
Nk1.1 APC/Lineage cocktail ETPs staining	FACS	0.25 μg/ml	eBioscience	Rat monoclonal Pk136
TCRγδ APC/Lineage cocktail ETPs staining	FACS	0.5 μg/ml	eBioscience	Rat monoclonal eBioGL3
CD11c APC/Lineage cocktail ETPs staining	FACS	0.25 μg/ml	eBioscience	Rat monoclonal HL3
Ter119 APC/Lineage cocktail ETPs staining	FACS	1 μg/ml	eBioscience	Rat monoclonal TER-119
B220 APC/Lineage cocktail ETPs staining	FACS	2 μg/ml	eBioscience	Rat monoclonal RA3-6B2
CD4 Alexa Fluor 700/ETPs staining	FACS	0.25 μg/ml	eBioscience	Rat monoclonal RMA-5
CD8a PEcy7/ETPs staining	FACS	0.25 μg/ml	eBioscience	Rat monoclonal 53.6.7
c-Kit APCef780/ETPs staining	FACS	0.25 μg/ml	eBioscience	Rat monoclonal 2B8
CD25 PerCP cy5.5/ETPs staining	FACS	2 μg/ml	eBioscience	Rat monoclonal PC61.5
**Secondary antibodies**				
Goat anti-Rabbit IgG (H+L) Secondary Antibody, Alexa Fluor 488	IF, FACS	1 μl/ml; 5 μl/ml	Thermo Scientific	R37116
Goat anti rabbit HRP	WB	0.3 μl/ml	Thermo Scientific	
Goat anti mouse HRP	WB	0.5 μl/ml	Thermo Scientific	
Protein A HRP	WB	1 μl/ml	CST	

Supplementary Table 3: The hairpin vectors used for CAML knockdown. The catalogue number, supplier, vector and hairpin sequences are listed.

Hairpin number	Catalog Number	Supplier	Vector Name
1	RMM4431-98920146	Dharmacon	pGIPZ
2	RMM4431-101228193	Dharmacon	pGIPZ
5	RMM4431-101232062	Dharmacon	pGIPZ

Hairpin Sequence
TGCTGTTGACAGTGAGCGACGTCTACTTCTTCACCTTCATTAGTGAAGCCACAGATGTAATGAAGGTGAAGAAGTAGACGCTGCCTACTGCCTCGGA
TGCTGTTGACAGTGAGCGCAAGCCACAGGACAGTGACAAATAGTGAAGCCACAGATGTATTTGTCACTGTCCTGTGGCTTTTGCCTACTGCCTCGGA
TGCTGTTGACAGTGAGCGAAGGGTAGTGCTTGGTGATTCATAGTGAAGCCACAGATGTATGAATCACCAAGCACTACCCTCTGCCTACTGCCTCGGA

Supplementary Table 4: The chemicals and drugs used, including manufacturer and working concentrations.

Chemicals	Manifacturer	Working concentration/dilution
BrdU labeling reagent (00-0103)	Thermofisher/Invitrogen	5 μl/ml
Akti1/2	TOCRIS	20 μM
666-15	TOCRIS	1 μM
Imatinib	CST	10 μM
