# Supplementary material for: Bi-directional signaling by membrane-bound KitL induces proliferation and coordinates thymic endothelial cell and thymocyte expansion
Source: Nat Commun. 2018 Nov 8;9:4685. doi: 10.1038/s41467-018-07024-0 (PMC6224562; doi:10.1038/s41467-018-07024-0)
Supplement: Supplementary file 3 — Description of Additional Supplementary Files [file 41467_2018_7024_MOESM3_ESM.pdf]

Description of Additional Supplementary Files

File Name: Supplementary Data 1
Description: Proteins with significantly altered phosphorylation between NIH3T3 cells incubated with Kit–Fc (N=3) and IgG-Fc (N=3) identified by mass spectrometry. 427 out of 2887 proteins with phosphorylation sites showed significant change. For each protein the UNIPROT accession number, protein description and systematic gene name, fold change in phospho-peptide abundance, and P-value (t-test) is shown.

File Name: Supplementary Data 2
Description: Ingenuity pathway analysis (IPA) of proteins with significantly altered phosphorylation from Supplementary Table 1. Pathways significantly enriched ($P<0.05$) are shown, as is the P-value for enrichment (as $-\log10(P)$), and the molecules associated with the pathway. For IPA analysis the default settings were used.
